# A Wearable System Composed of FBG-Based Soft Sensors for Trunk Compensatory Movements Detection in Post-Stroke Hemiplegic Patients

**DOI:** 10.3390/s22041386

**Published:** 2022-02-11

**Authors:** Daniela Lo Presti, Martina Zaltieri, Marco Bravi, Michelangelo Morrone, Michele Arturo Caponero, Emiliano Schena, Silvia Sterzi, Carlo Massaroni

**Affiliations:** 1 Research Unit of Measurements and Biomedical Instrumentation, Center for Integrated Research, Università Campus Bio-Medico di Roma, 00128 Roma, Italy; d.lopresti@unicampus.it (D.L.P.); m.zaltieri@unicampus.it (M.Z.); e.schena@unicampus.it (E.S.); c.massaroni@unicampus.it (C.M.); 2 Unit of Physical Medicine, Campus Bio-Medico di Roma, Rehabilitation of Policlinico Universitario, 00128 Roma, Italy; m.bravi@unicampus.it (M.B.); m.morrone@unicampus.it (M.M.); 3 ENEA Research Center of Frascati, 00044 Roma, Italy; michele.caponero@enea.it

**Keywords:** soft strain sensor, flexible wearables, fiber Bragg grating sensors, compensatory trunk movements detection, post-stroke hemiplegic patients

## Abstract

In this study, a novel wearable system for the identification of compensatory trunk movements (CTMs) in post-stroke hemiplegic patients is presented. The device is composed of seven soft sensing elements (SSEs) based on fiber Bragg grating (FBG) technology. Each SSE consists of a single FBG encapsulated into a flexible matrix to enhance the sensor’s robustness and improve its compliance with the human body. The FBG’s small size, light weight, multiplexing capability, and biocompatibility make the proposed wearable system suitable for multi-point measurements without any movement restriction. Firstly, its manufacturing process is presented, together with the SSEs’ mechanical characterization to strain. Results of the metrological characterization showed a linear response of each SSE in the operating range. Then, the feasibility assessment of the proposed system is described. In particular, the device’s capability of detecting CTMs was assessed on 10 healthy volunteers and eight hemiplegic patients while performing three tasks which are representative of typical everyday life actions. The wearable system showed good potential in detecting CTMs. This promising result may foster the use of the proposed device on post-stroke patients, aiming at assessing the proper course of the rehabilitation process both in clinical and domestic settings. Moreover, its use may aid in defining tailored strategies to improve post-stoke patients’ motor recovery and quality of life.

## 1. Introduction

Stroke is a leading cause of disability in adults worldwide [1,2], since 80% of survivors develop hemiparesis or severe movement limitation [3]. In particular, the impairment of upper limb motility affects up to 75% of stroke survivors, impeding the ordinary course of the most basic daily life activities [4]. To reduce the handicap extent, post-stroke medical management typically provides for the adoption of intensive rehabilitation programs based on different approaches aiming at restoring the damaged cerebral motor areas. Among others, upper extremities physiotherapeutic rehabilitation based on task-oriented training (such as grasping or reaching exercises) is, to date, the most used and successful one [5,6]. Unfortunately, the effectiveness of the motorial recovery path is often impaired by the so-called compensatory strategies, which are atypical movements involving different body districts, with the aim of balancing a reduced range of motions [7,8]. One of the most common compensation strategies adopted during upper limb rehabilitation is compensatory trunk movements (CTMs).

Several methods have been exploited to help physiotherapists identify and avoid CTMs. In this context, camera-based systems (such as motion capture systems (MoCap [9]) and visual feedback technologies [10]) have played a key role as they permit continuous monitoring of trunk kinematic patterns. Good results have also been obtained by the usage of robotic manipulators or exoskeletons [11,12]. However, the encumbrance of these devices leads to the need for dedicated spaces and complex setups, as well as high costs, and the requirement of specialized personnel have limited their use to laboratory scenarios or clinical settings only. On the contrary, wearable systems mainly based on inertial sensors have provided a less expensive and portable alternative to identify CTMs also in domestic environments [13,14,15,16,17,18]. Nevertheless, the trunk movement’s reconstruction is approximated to those retrieved by a few inertial units placed in equally few measurement nodes upon the user’s back. Furthermore, the application of rigid components broadly reduces the wearability of the system and may inhibit the natural movements’ pattern.

To provide a solution to the growing demand for flexibility, a wide range of materials, such as polymers, gels, or liquids [19,20], was proposed to develop wearable soft sensors. Nevertheless, only a few studies have documented their use for spinal monitoring purposes. In [21], Caviedes et al. proposed a flexible triangular sensor array to monitor the spine posture and, thus, the correctness of task execution in postural rehabilitation therapies. However, the system did not allow for the detecting of CTMs. In [22], Kim et al. introduced a full body suit that integrated 20 soft sensors based on a liquid metal compound dedicated to body movements’ monitoring by means of deep learning algorithms. Although six sensors were distributed on the back, no sensors were positioned along the spine. 

Thanks to their high flexibility, small size, and light weight [23,24], fiber optic sensors based on fiber Bragg grating (FBG) technology have proven to be easily embedded in soft matrices for the development of flexible sensors. Such sensors have been largely employed to design smart wearables for the detection of physiological parameters, such as respiratory frequency [25,26,27,28,29], heart rate [30,31,32], and joint movements [33,34,35]. In [34,36,37,38], our research team assessed the feasibility of a single FBG-based soft sensor to detect flexion/extension back movements, including the cervical spine. However, the proposed devices were not suitable to detect CTMs.

In this work, we present a lightweight and comfortable wearable system for CTMs’ detection composed of seven modular soft sensing elements (SSEs) based on FBG sensors. FBGs’ features of high strain sensitivity (*Sε*) and multiplexing capability [23,24] enable reliable and multi-point monitoring of trunk movements for CTMs’ detection. Indeed, skin deformation caused by trunk movements leads to a change in the SSEs’ output, thus allowing trunk recruitment quantification. The use of the proposed device may lay the basis for monitoring the proper course of post-stroke patients’ motor recovery in a home environment. Moreover, its exploitation may help in defining patient-tailored strategies to improve the rehabilitation process.

## 2. The Wearable System: Working Principle, Manufacturing, and Metrological Assessment

In this section, we firstly described the FBG principle of work. Then, we focus on the main steps of the manufacturing process, from the system design to the fabrication. Finally, we investigate the response to applied strain and temperature variation of each SSE. 

### 2.1. FBG’s Working Principle

An FBG is a resonant structure inscribed into the core of an optical fiber, where the core refractive index is modulated to create a grating which works as a stop-band filter. In fact, once illuminated by a broad band spectrum light sourced by an optical interrogator, most of the light passes through the grating, except for a small amount that is back-reflected. The reflected light is centered at the Bragg wavelength (*λ_B_*), which is different for each FBG inscribed along the same fiber, to avoid any overlapping of the gratings’ responses. The *λ_B_* depends on the fiber core effective refractive index (*η_eff_*) at the grating and the grating period (Λ), as defined in (1):

(1) $$\lambda_{B}=2\cdot \eta_{eff}\cdot \Lambda$$

Temperature variations (Δ*T*) and strain (ε) produce changes in *η_eff_* and Λ, causing a shift of *λ_B_* (Δ*λ_B_*):

(2) $$\frac{\Delta \lambda_{B}}{\lambda_{B}}=\left(1-p_{e}\right)\cdot \varepsilon +\left(\left(1-p_{e}\right)\cdot \;\alpha +\xi \right)\cdot \Delta T$$

where p_e_ represents the photoelastic coefficient, α is the thermal expansion coefficient, and ξ denotes the thermo-optic coefficient [39]. 

Focusing on our application, when each SSE is placed in contact with the back, any deformation occurring on the skin surface is directly transmitted to the polymer matrix and, in turn, to the encapsulated FBG, according to bonding strengths at the matrix–optical fiber interface. As a consequence, trunk displacements produce variations in the FBG output, resulting in Δ*λ_B_*. Instead, the Δ*T* contribution on the SSEs’ outputs may be deemed negligible. This aspect is discussed in detail in Section 2.3.2.

### 2.2. Design and Manufacturing Process

The wearable system is a 1 m-length multi-point sensing device designed to be placed along the back spine.

**Figure 1:**
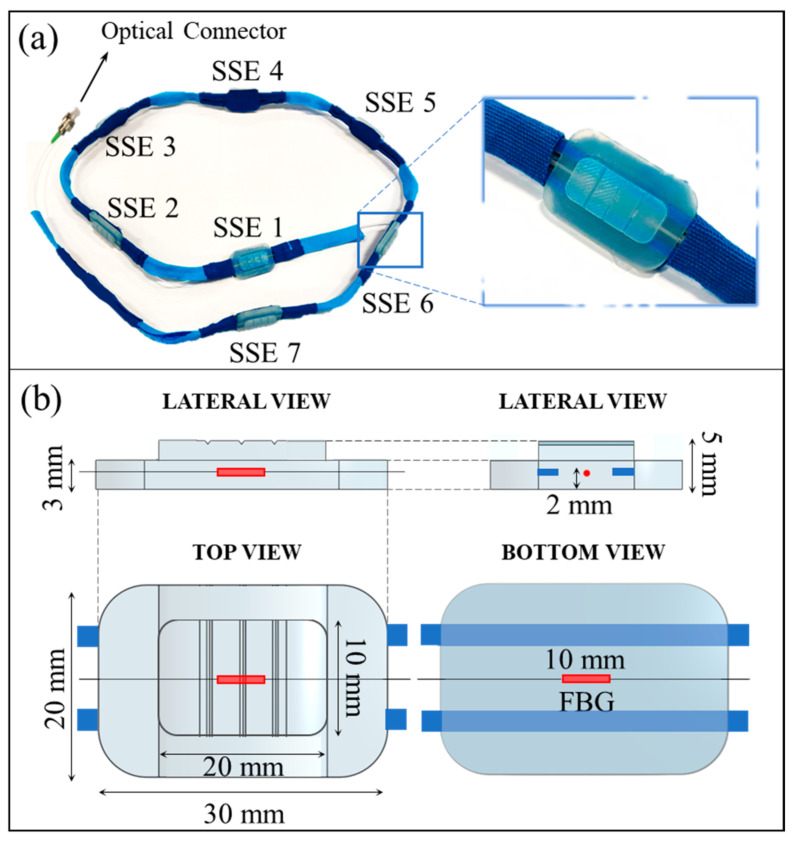
The wearable system. (**a**) A picture of the wearable system (on the left), together with a close-up view of one of the seven identical sensing elements (on the right); (**b**) Rendering and features of the sensing elements shown in lateral, top, and bottom views.

The device consists of a commercial FBG array with seven multiplexed gratings (*λ_B_* values ranging from 1512 nm to 1559 nm, gratings’ length of 10 mm, and reflectivity of 90%, AtGrating Technologies, Shenzhen, China), interspaced 100 mm from each other with an FC/APC optical connector at the fiber end. Each FBG is enclosed into a soft silicon matrix (Dragon SkinTM30, Smooth-On, Inc., Macungie, PA, USA) to comply with the physiological back curvature. As a result, seven identical SSEs were developed, named SSE1, SSE2, SSE3, SSE4, SSE5, SSE6, and SSE7, from the farthest to the nearest to the optical connector (see Figure 1a). 

Each SSE (whose rendering and features are reported in Figure 1b) comes as a structure formed by two superimposed polyhedra with rectangular bases and rounded corners. The lower polyhedron (30 mm × 20 mm × 3 mm) contains an FBG placed at 2 mm from the bottom in its central section. This portion provides a wide adherence area to ensure reasonable compliance with the skin, as it is intended to be bonded to the subject’s back through biocompatible double-sided tape. A smaller polyhedron (20 mm × 10 mm × 2 mm) presenting three parallel grooves (0.4 mm deep) on its surface is placed above the FBG and centered to the lower polyhedron. Such extrusion both strengthens the sensor by preventing fiber damages caused by excessive traction in the central area and avoids undue deformations by distributing the surface tensile stress by means of the grooves. The fiber portion between two consecutive SSEs is held in small flexible plastic tubes (eight tubes in total). The small tubes improve the robustness of the portions of the optical fiber that are not encapsulated into the silicone matrices. Moreover, these portions allow the sensor’s arrangement at specific anatomical landmarks, thus respecting inter-subject anthropometric variability.

The fabrication process of the proposed wearable systems required the following main steps:

1. The design and 3D-printing of seven molds;
2. The FBG’s positioning at the molds’ midsection before the gratings’ encapsulation into the flexible matrices;
3. The polymer reticulation by mixing part A and part B of a bi-component silicone rubber;
4. The mixture pouring into the molds and curing for 24 h at room temperature before pulling out each SSE from the mold.

**Figure 2:**
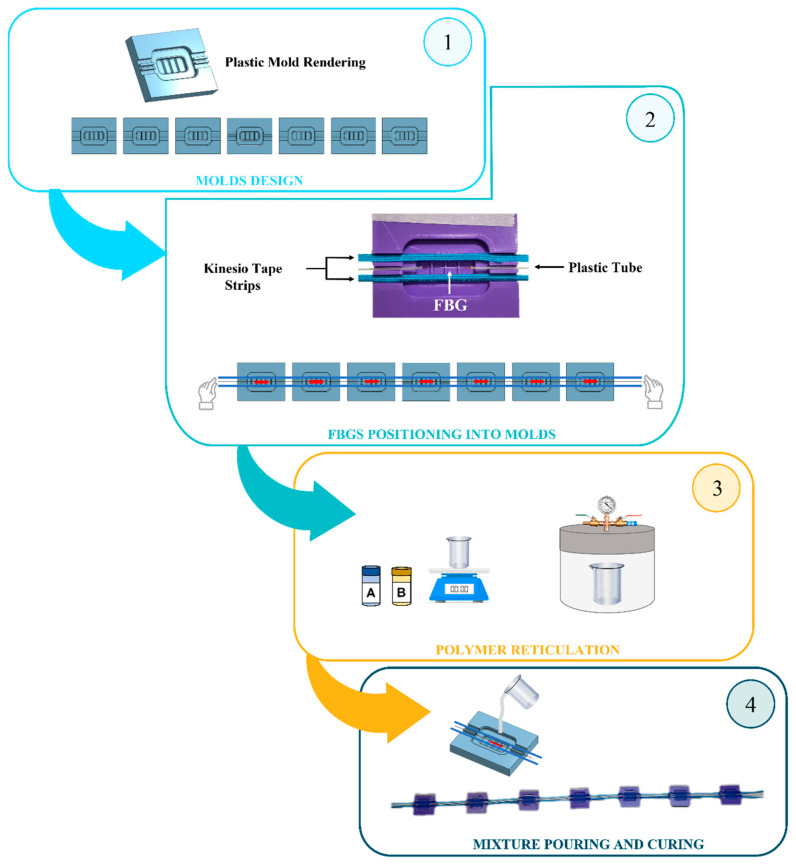
Diagram showing the manufacturing process of wearable system.

The main steps of the fabrication process are represented in Figure 2. Further details regarding the manufacturing of the wearable system are given in Section 1 of the Supplementary Material and shown in Figure S1.

### 2.3. Response to Strain and Temperature Influence

In the present study, the proposed wearable system is intended to be used as a strain sensor for detecting the back movements of the enrolled subjects. Hence, a metrological characterization was performed to estimate the Sε of each SSE. Although the experiments were carried out at a constant room temperature (T), once the flexible matrices have been attached to the skin, slightly changes of T (*ΔT*) may occur at the skin–matrix interface. Therefore, the SSEs’ responses to *ΔT* were also investigated. 

#### 2.3.1. The Sensing Elements’ Response to Strain

Compared to a bare FBG, the presence of a polymeric structure can lead to variations in the sensor response to ε [40]. In fact, a reduction of *Sε* value is expected, since ε is not directly applied on the grating but on the polymer matrix and then transmitted to the encapsulated FBG. Therefore, the response to ε of the SSEs was evaluated by using a tensile testing machine (Instron 3365A, Instron, Norwood, MA, USA) at quasi-static conditions (i.e., 2 mm·min^−1^ of elongation speed) and room temperature. 

**Figure 3:**
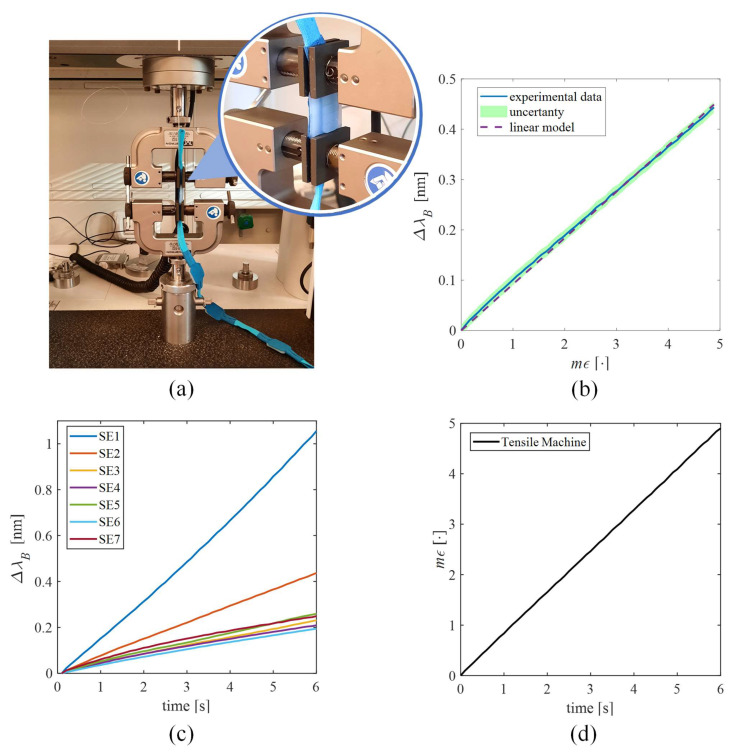
Response to strain of the SSEs. (**a**) The testing machine and a zoom of the sensing element placed between the two clampers. (**b**) The calibration curve of ΔλB vs. mε of SSE2, given as an example. The mean experimental Δλ_B_ signal is shown by the blue line, the uncertainty is shown by the shadowed green area, and the linear model is shown by the dotted purple line. (**c**) The average Δ*λ_B_* vs. time of all the SSEs. (**d**) The strain vs. time.

Each SSE was positioned between the machine clampers and fixed to the edges of the polymeric substrate (see Figure 3a). 

The SSE was strained nine times (to ensure event repeatability) from 0% to 0.5% of *ε* with respect to its initial length to cover the ε range that could be experienced by the sensor in response back movements. Values of ε were collected at a sampling frequency of 10 Hz, while the FBGs’ outputs (i.e., *Δλ_B_*) were recorded by an optical interrogator (si255 Hyperion Platform, Micron Optics Inc., Atlanta, GA, USA) at 100 Hz. Data processing was executed in the MATLAB^®^ environment (MathWorks^®^ Inc., Natick, MA, USA). For each SSE, the mean value of Δ*λ_B_* and the expanded uncertainty were calculated across the nine tests. In Figure 3c,d, the average trends of Δ*λ_B_* vs. time for each SSE across the nine tests and the applied strain are reported, respectively. The expanded uncertainty was obtained as the standard uncertainty multiplied by the coverage factor (k = 2.30), considering a t-student distribution with eight degrees of freedom and a confidence level of 95% [41]. The calibration curve was estimated as the best fitting line of the average *Δλ_B_* over ε and Sε as the slope of the best fitting line. The high values of correlation coefficients (R^2^ > 0.99 for all of the SSEs’ calibration curves) ensured agreement between the experimental data and the linear model. 

Figure 3b shows the calibration curve of a single SSE (i.e., SSE2), while the *Sε* values of all the SSEs are listed in Table 1. The different Sε numerical values are attributable to the fabrication process, which is manually executed. In fact, although the manufacturing was carried out with the utmost carefulness, it cannot be excluded that slight differences in the tensioning of the fiber during its positioning into molds and variations in the bonding strength at the fiber–polymer matrix interface during the curing might have occurred in each SSE. For the calibration curves of Δ*λ_B_* vs. *mε* of all SSEs, please refer to Section 2 of the Supplementary Material, Figure S2. 

#### 2.3.2. The Sensing Elements’ Response to Temperature

**Figure 4:**
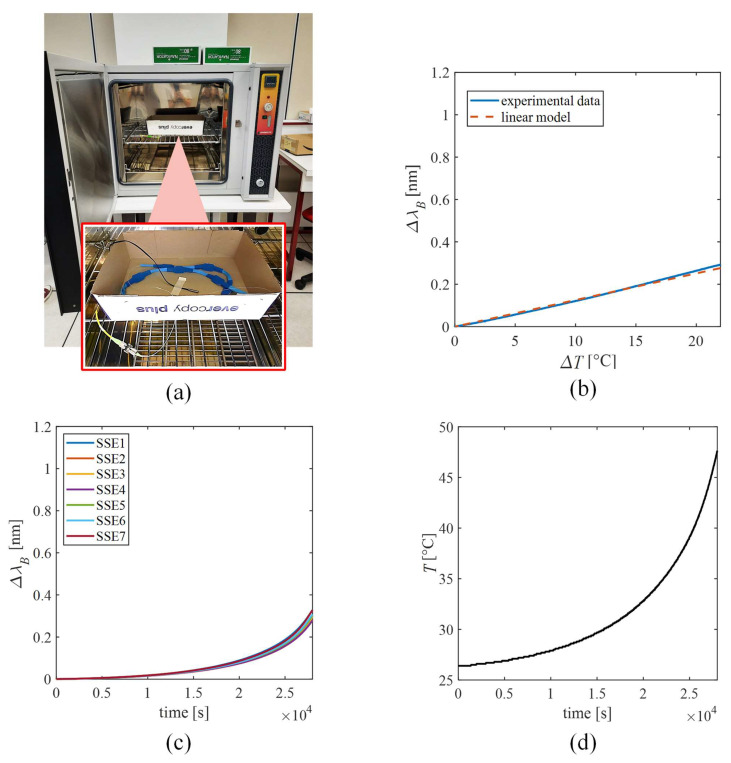
Response to temperature of the SSEs. (**a**) The laboratory oven and a zoom of the wearable system placed inside the oven. (**b**) The calibration curve of *Δλ_B_* vs. *ΔT* of SSE2 is given as an example. The experimental *Δλ_B_* signal is shown as the blue line, while the linear model is shown as the dotted purple line. (**c**) The Δ*λ_B_* vs. time of all the SSEs. (**d**) The temperature vs. time.

Considering the FBG intrinsic sensitivity to *T* (*S_T_*), the response of SSEs to *ΔT* was also investigated. The influence of *T* on the SSEs’ output was evaluated by placing the wearable system within a laboratory oven (PN120 Carbolite^®^), as shown in Figure 4a, and exposing it to T changes from 26 °C to 50 °C to extensively cover the working range of SSE. Reference values of T were collected by a thermistor (EL-USB-TP-LCD, EasyLog, Lascar Technology) and the output of SSEs by the FBG interrogator (FS22, HBM FiberSensing). A sampling frequency of 1 Hz was set for both the devices. The trends of SSEs’ output changes and *T* over time are shown in Figure 4c,d, respectively All of the *Δλ_B_* values ranged from ~0 nm up to 0.43 nm when exposed to *T* ranging between ~26 °C to ~49 °C. To extract the calibration curve (see Figure 4b), the Δ*λ_B_* of each SSE was plotted against *ΔT* (blue line), and the best fitting line was computed (dotted orange line). The S_T_ value of each SSE was obtained as the slope of the best fitting line and listed in Table 2. 

Our findings showed S_T_ values similar to a bare FBG (i.e., 0.01 nm·°C ^−1^) and a negligible influence of *T* on the SSEs’ output during the experimental phase. Therefore, no changes in the thermal expansion of the polymer matrices occurred. Indeed, in each trial (described in the following paragraph), typical every-day life tasks were replicated. The whole test sequence lasted about 20 min per patient. Therefore, the thermal stability at the matrix–body interface due to the body thermoregulation, as well as the smooth environmental *ΔT*, which may occur in the scenario of interest, led to a negligible T influence on the SSE output. For the calibration curves of *Δλ_B_* vs. ΔT of all SSEs, please refer to Section 3 of the Supplementary Material, Figure S3. 

## 3. Feasibility Assessment of the Wearable System in CTMs’ Detection

In this section, a preliminary assessment of the wearable system on healthy subjects is reported. Then, the experimental trials conducted on hemiplegic patients are presented.

### 3.1. Experimental Trial on Healthy Volunteers

An explorative test was performed on 10 healthy volunteers (whose features are reported in Table 3), performing tasks employing the upper limb to inquire about the wearable system’s capability to detect CTMs. All subjects read and signed informed consent. The study protocol was approved by the Ethics Committee of Università Campus Bio-Medico di Roma (protocol code ST-UCBM 27.2(18).20 OSS) and in accordance with the guidelines of the Declaration of Helsinki. 

**Figure 5:**
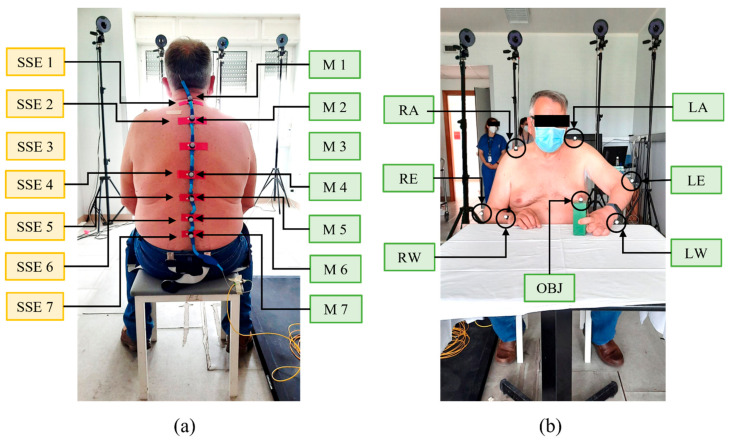
Soft sensing elements’ and markers’ positioning on a volunteer. In (**a**) the placement of the seven sensors (yellow box texts) and markers (green box texts) on the back of the volunteer is shown. In (**b**) the placement of the seven markers (green box texts) upon the object and the volunteer’s acromia, elbows and wrists is shown.

Participants were invited to sit on a stool, bare-chested or (in the case of female subjects) wearing a track top. The wearable system was applied on the back of each subject with hypoallergenic biocompatible tape on the user-facing side. SSE5 was placed on the T12 vertebra, which identifies the point of inversion of the thoracic and lumbar curves, thus subdividing the back into upper and lower portions. SSE1 and SSE7 were then placed on the C7 and L3 vertebrae, respectively. Then, SSE2, SSE3, and SSE4 were equidistantly placed between SSE1 and SSE5, while SSE6 was fixed between SSE5 and SSE7. All sensors were then secured to the skin with an extra piece of Kinesio tape that was designed to stay in place all day, also during sweating. A stereophotogrammetric MoCap (BTS D-Smart, by BTS Bio-Engineering S.r.l., Milan, Italy) was used to quantitively measure the tridimensional movements of the upper part of the participants’ body. Four cameras were installed behind the seated subject at ~2 m, and four were in front of the volunteer. Fourteen spherical markers (12 mm in diameter) attached with hypoallergenic tape were used and placed upon each SSE (M1, M2, M3, M4, M5, M6, and M7), on the right and left acromia (RA and LA, respectively), elbows (RE and LE, respectively) and wrists (RW and LW, respectively) and on the moving object (OBJ). The positioning of SSEs and markers is depicted in Figure 5. 

**Figure 6:**
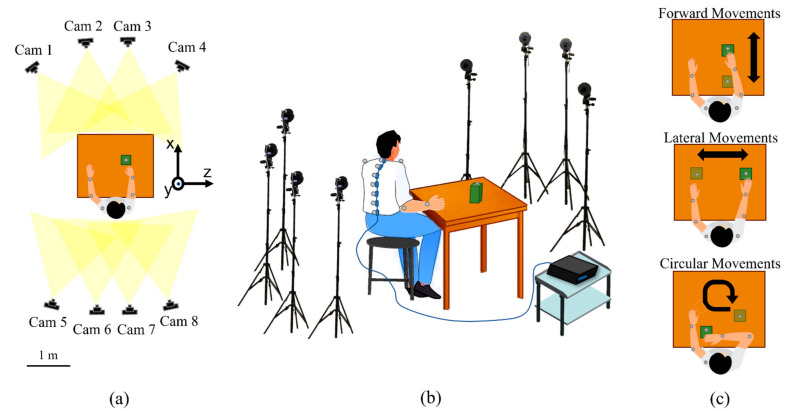
Experimental setup and protocol. (**a**) Upper view showing the positioning of the eight cameras and the reference axes. (**b**) The experimental set-up showing the subject’s positioning, the MoCap system, the wearable system, and the spectrum interrogator. (**c**) Illustration of the three tasks performed during the protocol.

Furthermore, in Figure 6a,b, the experimental setup is represented.

Participants were instructed on the experimental protocol, which consisted in sitting at a table while wearing the wearable system and moving an object (i.e., wooden polyhedron of 10 cm × 15 cm × 5 cm) across the table’s surface. The following three tasks (see Figure 6c), which are representative of typical everyday life actions, were performed:

1. Forward Movements (FM): move the object back and forth by executing a flexion–extension movement of the arm;
2. Lateral Movements (LM): move the object right and left, keeping the arm outstretched;
3. Circular Movements (CM): move the object, performing circular motions.

For each of the three tasks, all participants performed two rounds (i.e., Round 1 and Round 2), each consisting of 10 repetitions using the dominant arm. In Round 1, the volunteers executed the repetitions by avoiding the trunk recruitment, while in Round 2, the subjects performed the same repetitions by eliciting CTMs which typically occur in the presence of hemiplegia. More specifically, in Round 2, each volunteer was invited to self-maintain a stable upper limb pose while performing the task, to promote trunk involvement (in line with [14]).

During the protocol execution, the wearable system’s data were collected by means of an optical interrogator (si255, Micron Optics Inc., Atlanta, GA, USA) at a 1 kHz sampling rate, while the 3D markers’ trajectories were recorded with a BTS Tracker software (by BTS Bioengineering S.r.l., Milan, Italy) at a 60 Hz sampling rate.

**Figure 7:**
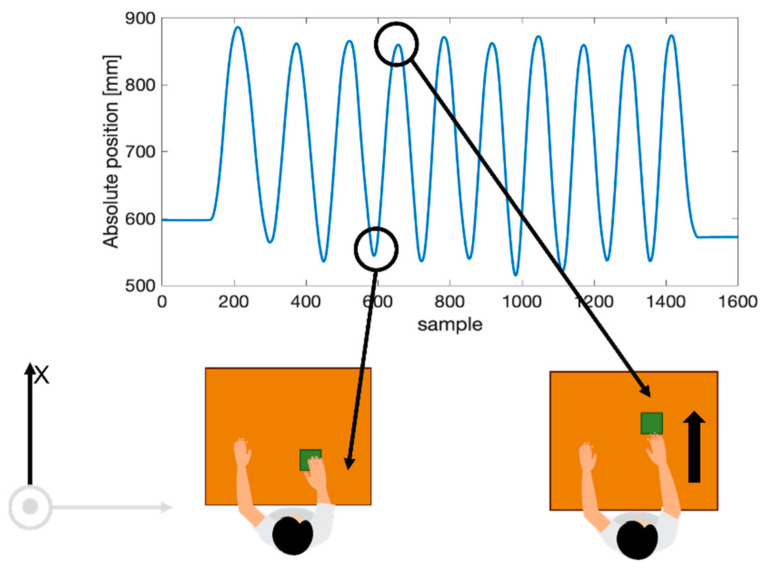
Example of the movement of the object along the x-axis. The highlighted minimum point represents the instant of the minimal distance of the object from the subject, while the highlighted maximum point represents the instant of the maximal distance from the subject.

Experimental data were processed in the MATLAB^®^ environment (MathWorks^®^ Inc., Natick, MA, USA). MoCap was used as a reference instrument to determine the magnitude of the trunk involvement and, in turn, the presence of CTMs. Raw trajectories of all the markers in all the planes (sagittal plane x–y, transversal plane y–z, and frontal plane x–z) were recorded. In each trial, the marker positioned on the object was used to subdivide the recorded trace into 10 signals related to the performed movements. Specifically, Figure 7 shows the periodic movement on a single axis (x) of the object during the 10 consecutive movements executed by a user. The movement of the object is a periodic signal. The minimum points represent the instants in which the object was at the minimum distance from user, while the maximum points are those in which the object was at the maximum distance from the user. A complete movement was considered as the signal between two consecutive minima points. As a consequence, 10 movements per each trial were identified, and the seven markers’ trajectories on the x-, y-, and z-axes were segmented into 10 windows. For each window, the relative marker displacement amplitude was calculated as the difference between the max value and the min value of the displacement. Hence, 10 values were obtained per each marker and per each axis considering each trial. The median value over the 10 movements was calculated to obtain the median relative displacement (*R*) of all the markers along x (*Rx*), y (*Ry*), and z (*Rz*) directions separately. The *Rx*, *Ry*, and *Rz* were considered an index of trunk displacement along the three axes: the higher the value of *R*, the higher the trunk involvement.

**Figure 8:**
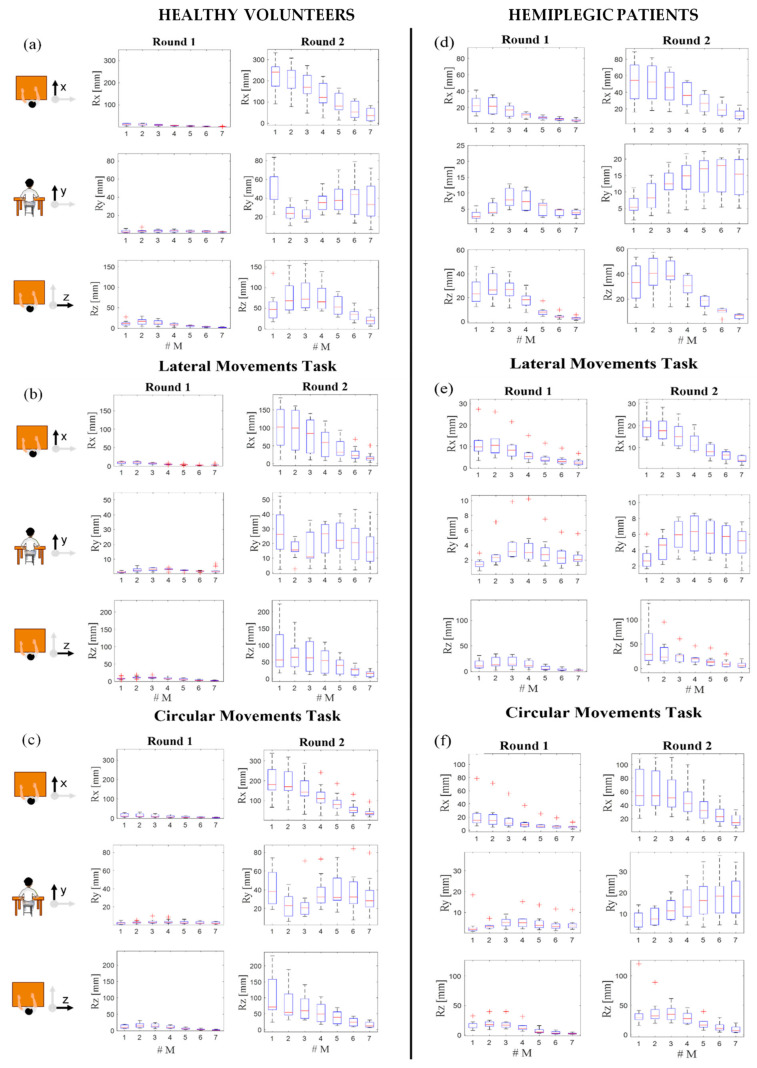
Relative displacements of the 7 MoCap markers (#M) placed on the spline along *x* (*Rx*)-, *y* (*Ry*)-, and *z* (*Rz*)-axes during the three tasks (forward movements in (**a**) and (**d**), lateral movements in (**b**) and (**e**), and circular movements in (**c**) and (**f**)), performed by the healthy volunteers (left column) and hemiplegic patients (right column), in the presence (Round 1) and absence (Round 2) of CTMs. Data are expressed as median, IQRs, and outliers.

Figure 8 (left column) shows the box plots obtained by grouping all the *R* data gathered by different subjects. In each box plot, the median and the interquartile range (IQR) quantify the magnitude and inter-subject variability of the trunk involvement. As expected, *Rx*, *Ry*, and *Rz* values corresponding to each spinal marker were always greater in Round 2 (presence of CTMs) than in Round 1 (without CTMs). In all the tasks, the median values of *R* registered from the seven markers were approximately one order of magnitude greater than those in Round 1 in all the axes. Moreover, it is worth noting that during the FM and LM tasks, larger *Rx* and *Rz* values occurred, respectively. The lowest values were related to *Ry* in all tasks. Those registered from the seven markers were approximately one order of magnitude greater than those in Round 1 in all tasks.

From the output of each SSE, the ε trends in time were retrieved for all the SSEs as follows:

(3) $$\varepsilon =\frac{\Delta \lambda_{B}}{S_{\varepsilon }}$$

**Figure 9:**
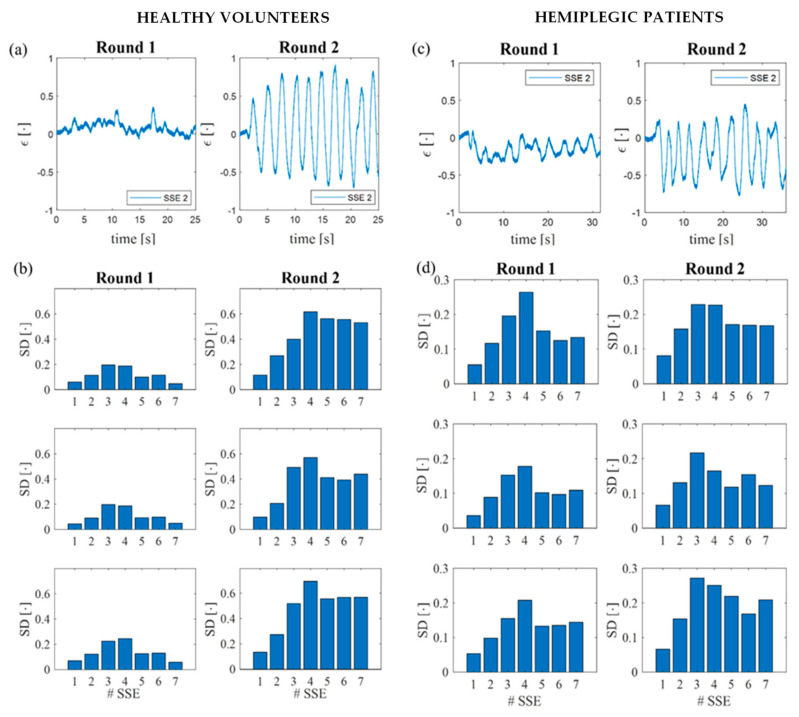
Example of the ε trends in time retrieved by SSE 2 during the execution of the CM task performed by a healthy volunteer (**a**) and a hemiplegic patient (**c**) in Round 1 and Round 2. Mean SD values calculated for the 7 SSEs (# SSE) during the three tasks performed by the healthy volunteers (**b**) and hemiplegic patients (**d**) in Round 1 and Round 2.

In Figure 9a, an example of the output obtained by one of the seven SSEs is reported. To quantify the *ε* experienced by each SSE during trunk displacements, for every subject, we evaluated the standard deviation (SD) over the 10 repetitions of each task. Then, for each SSE and task, we calculated the mean value of the SDs across the 10 healthy volunteers. In Figure 9b, the mean SDs of SSEs (from 1 to 7) are reported for the three tasks considering both Rounds 1 and 2. In all the trials, each SSE showed higher SD values in Round 2 than in Round 1, confirming the presence of CTMs.

### 3.2. Experimental Trial on Hemiplegic Patients

Having preliminarily verified the system’s capability to detect CTMs on healthy volunteers, an assessment was performed on post-stroke hemiplegic patients. Participants were recruited from a research volunteer database produced by the Physical and Rehabilitation Unit of Fondazione Policlinico Universitario Campus Bio-Medico. A total of 8 hemiplegic patients (whose features are shown in Table 4) were enrolled. Inclusion criteria were (i) acquired diagnosis of stroke, (ii) absence of cognitive deficits, and (iii) a score of the Fugl-Meyer Assessment Upper Extremity Scale of Motor Impairment (i.e., the most used rating scale to measure post-stroke disability extent [42]) ≥ 10 [43]. This value was identified as the minimum value to guarantee residual upper limb functionality to perform the required tasks.

Hemiplegic patients were instructed to perform the same experimental protocol executed by the healthy volunteers but, this time, using the unaffected arm in Round 1 and the hemiplegic arm in Round 2. The Rx, Ry, and Rz medians and the IQRs values obtained by the MoCap data are shown in Figure 8 (right column). As for the healthy volunteers, the Rx, Ry, and Rz values corresponding to each marker (from M1 to M7) during the three tasks were always higher in Round 2 (i.e., affected arm and presence of CTMs) than in Round 1 (i.e., unaffected arm and absence of CTMs). As before, it is possible to state that the lowest values were related to Ry in all the tasks. Instead, during the execution of FM and LM tasks, larger Rx and Rz values occurred, respectively.

Regarding the wearable system, ε trends in time were obtained for all SSEs (in Figure 9c, an example is given). In Figure 9d, the SDs calculated for all the SSEs are reported for the three tasks grouped in Rounds 1 and 2. Each SSE showed higher mean SDs in Round 2 than in Round 1, considering all the three tasks, except for the values obtained from SSE4 during the performance of FM and LM tasks. In fact, SSE4 presented mean SD values of 0.264 and 0.178 in Round 1 of FM and LM, respectively, and of 0.227 and 0.165 in Round 2 for FM and LM, respectively.

## 4. Discussion and Conclusions

This study presents a novel wearable system composed of seven FBG-based SSEs for CTMs’ detection in post-stroke upper limbs patients. The manufacturing of the wearable device and its response to ε were described. The ability of the proposed device to detect CTMs was firstly proven on 10 healthy volunteers in the presence and absence of trunk recruitment and then on eight hemiplegic patients using the affected and unaffected arms. 

MoCap was used as a benchmark of trunk involvement. Data provided by markers produced median R values ranging from 1 mm to 25 mm in Round 1 of each task performed by the healthy population (Figure 8, left column). The comparison of these data with the ones recorded on hemiplegic patients showed slightly higher values for the tasks performed with the unaffected arm (i.e., between 1 mm and 33 mm) and a more relevant R value increment for the ones executed with the hemiplegic arm (i.e., up to 90 mm), as reported in Figure 8, in the right column. These results confirm the presence of CTMs in the Round 2 performed by the hemiplegic population.

In accordance with the MoCap, the wearable system demonstrated its ability in detecting CTMs. In fact, comparable mean SD values (i.e., between 0.1 and 0.25) were retrieved for Round 1 performed by both the healthy and the hemiplegic populations (see Figure 9b,d, respectively), while higher mean SD values were retrieved for the tasks performed by hemiplegic patients by means of the affected arm (i.e., Round 2 shown in Figure 9d) and in the presence of CTMs. These results are in agreement with the MoCap data. Nevertheless, an exception was represented in FM and LM tasks of Round 2 performed by the hemiplegic patients, since SSE4 produced higher mean SD values than the ones obtained for Round 1 both for the healthy (Figure 9b) and hemiplegic volunteers (Figure 9d). This finding may have been related to the SS4 position on the back. In fact, as shown in Figure 5, SSE4 was in contact with the area of the back in which the spine changes its concavity. Therefore, this condition may have affected its adhesion to the skin during the execution of tasks, lowering the sensor performances. Nonetheless, the exploited multi-sensor approach enables one to detect the presence of CTMs, even if one sensor shows poor reliability (as for SSE4).

The development of the proposed wearable device was stimulated by the growing interest in the use of novel wearable technologies in rehabilitation scenarios, where portability, low bulkiness, good accuracy, and affordable costs are mandatory. In the context of the post-stroke upper extremities’ rehabilitation, inertial sensors have been largely exploited [13,14,15,16,17,18]. However, most of these systems manifested inaccuracy in detecting slow movements [44]. This limit is a major drawback, since all post-stroke rehabilitation processes are aimed at individuals with incipient motor impairment, which largely reduces the speed of tasks’ execution. On the contrary, the presented wearable system is based on FBG technology showing a high sensitivity and good frequency response, even when encapsulated in polymeric matrices [24,45]. A demonstration was given by the capability of the wearable system to perceive even the smallest trunk deformations during the exercises performed by hemiplegic patients.

A second issue given by the usage of a limited number of inertial sensors is the lack of a high spatial resolution in the trunk displacements’ reconstruction. In fact, no more than two inertial units have been used for this aim [13,14,15,16,17,18], and, as a result, trunk movements are approximated to those of the sole sensors’ placement. In contrast, the proposed wearable system is composed of multiple, freely placeable SSEs that enable measurements at different spine levels. 

A further drawback apported by the usage of inertial sensors is the application on the back of rigid and uncomfortable components that often inhibit free movements’ execution. To address this problem, technological research has increasingly moved toward the development of soft, highly flexible, bendable sensors. Several soft sensors have been proposed for monitoring different joint angles, but only a few have been proposed specifically for the back area [21,22,36,37,38]. However, none of these are intended to detect CTMs. Consequently, the presented wearable system is the first one based on multiple soft sensors distributed along the whole spine, whose purpose is to evaluate the trunk recruitment.

Although the findings of this study are promising, further investigation will be devoted to the enlargement of the sample size. It is worth mentioning that the experiments were conducted on a small sample of patients, covering a wide range of Fugl-Meyer Assessment Upper Extremity scores (from 11 up to 55). The investigation of other statistical indices and features will be addressed to strengthen our results in terms of CTMs’ detection.

In a future perspective, the proposed system will help in evaluating the post-stroke recovery for optimizing the rehabilitation path. In addition, the constant advancement of technology in the development of ever smaller, lighter, and more portable fiber-optic interrogation units could open up a path for a portable approach for FBG-based devices. Therefore, the presented wearable system could be not only employed in clinical environments, but also in domestic scenarios, thus ensuring all-round patient support and monitoring during all the rehabilitation activities. 

## Figures and Tables

**Table 1:** Values of the. Sε obtained for the seven SSEs.

# SSE.	Sε [nm·mε^−1^]
SSE1	0.21
SSE2	0.09
SSE3	0.05
SSE4	0.05
SSE5	0.06
SSE6	0.04
SSE7	0.06

**Table 2:** Values of the. S_T_ obtained for the seven SSEs.

# SSE	S_T_ [nm·°C^−1^]
SSE1	0.015
SSE2	0.013
SSE3	0.012
SSE4	0.013
SSE5	0.014
SSE6	0.014
SSE7	0.015

**Table 3:** Features of the healthy volunteers.

# Volunteer	Age [y.o.]	Sex	Dominant Hand	Height [cm]	Body Mass [kg]
1	26	Female	Right	168	52
2	24	Female	Right	165	60
3	23	Male	Right	179	72
4	28	Male	Right	177	76
5	24	Female	Right	154	48
6	22	Male	Right	186	81
7	30	Female	Right	170	60
8	32	Male	Right	163	61
9	20	Male	Right	180	72
10	23	Female	Right	160	52

**Table 4:** Features of the hemiplegic patients.

# Patient	Age [y.o.]	Sex	Affected Side	Height [cm]	Body Mass [kg]	UE-FMA *
1	55	Male	Left	170	74	11
2	73	Male	Left	175	106	37
3	63	Male	Right	175	88	43
4	33	Female	Right	168	54	55
5	63	Male	Left	170	79	32
6	47	Male	Left	184	86	24
7	55	Male	Right	168	49	50
8	43	Male	Left	165	75	34

* Fugl-Meyer Assessment Upper Extremity score.

## Data Availability

The data presented in this study are available on request from the corresponding author. The data are not publicly available due to privacy restrictions.

## Supplementary Materials

The following supporting information can be downloaded at: https://www.mdpi.com/article/10.3390/s22041386/s1: Section 1 “Wearable System Fabrication”: Insight into the manufacturing process of the wearable system; : Representation of the eight steps composing the manufacturing process of the wearable system; Figure S2: The calibration curve of Δλ_B_ vs. mε of the seven SSEs: (**a**) SSE 1, (**b**) SSE 2, (**c**) SSE 3, (**d**) SSE 4, (**e**) SSE 5, (**f**) SSE 6 and (**g**) SSE 7. The mean experimental Δλ_B_ signal is shown by the blue line, the uncertainty is shown by the shadowed green area, and the linear model is shown by the dotted purple line; Figure S3: The calibration curve of Δλ_B_ vs. ΔT of the seven SSEs: (**a**) SSE 1, (**b**) SSE 2, (**c**) SSE 3, (**d**) SSE 4, (**e**) SSE 5, (**f**) SSE 6 and (**g**) SSE 7. The experimental Δλ_B_ signal is shown as the blue line, while the linear model is shown as the dotted purple line.
