# Supplementary material for: A Wearable System Composed of FBG-Based Soft Sensors for Trunk Compensatory Movements Detection in Post-Stroke Hemiplegic Patients
Source: Sensors (Basel). 2022 Feb 11;22(4):1386. doi: 10.3390/s22041386 (PMC8963020; doi:10.3390/s22041386)
Supplement: Supplementary file 1 [file sensors-22-01386-s001.zip › sensors-1580593-supplementary.pdf]

sensors

MDPI

Supplementary Material

A Wearable System Composed of FBG-Based Soft Sensors for Trunk Compensatory Movements Detection in Post-Stroke Hemiplegic Patients

Daniela Lo Presti [1,†], Martina Zaltieri [1,†], Marco Bravi [2], Michelangelo Morrone [2], Michele Arturo Caponero [3], Emiliano Schena [1], Silvia Sterzi [2,*] and Carlo Massaroni [1]

[1] Research Unit of Measurements and Biomedical Instrumentation, Center for Integrated Research, Università Campus Bio-Medico di Roma, 00128 Roma, Italy; d.lopresti@unicampus.it (D.L.P.); m.zaltieri@unicampus.it (M.Z.); e.schena@unicampus.it (E.S.); c.massaroni@unicampus.it (C.M.)
[2] Unit of Physical Medicine and Rehabilitation of Policlinico Universitario Campus Bio-Medico di Roma, 00128 Roma, Italy; m.bravi@unicampus.it (M.B.); m.morrone@unicampus.it (M.M.)
[3] ENEA Research Center of Frascati, 00044 Roma, Italy; michele.caponero@enea.it
* Correspondence: s.sterzi@unicampus.it
† These authors contributed equally to this work

Citation: Presti, D.L.; Zaltieri, M.; Bravi, M.; Morrone, M.; Caponero, M.A.; Schena, E.; Sterzi, S.; Massaroni, C. A Wearable System Composed of FBG-Based Soft Sensors for Trunk Compensatory Movements Detection in Post-Stroke Hemiplegic Patients. *Sensors* **2022**, *22*, 1386. https://doi.org/10.3390/s22041386

Academic Editor: Shyqyri Haxha

Received: 17 January 2022
Accepted: 9 February 2022
Published: 11 February 2022

Publisher's Note: MDPI stays neutral with regard to jurisdictional claims in published maps and institutional affiliations.

Copyright: © 2022 by the authors. Licensee MDPI, Basel, Switzerland. This article is an open access article distributed under the terms and conditions of the Creative Commons Attribution (CC BY) license (https://creativecommons.org/licenses/by/4.0/).

1. Wearable System Fabrication

The wearable system is a multi-point sensing system composed of seven flexible soft sensing elements (SSEs) based on FBG technology.

The steps followed to manufacture the wearable system are detailed below:

I. Seven identical plastic molds created with a design software (OnShape®, PTC, Boston, MA, USA) were 3D printed by Ultimaker 2+ (Ultimaker B.V., Utrecht, The Netherlands). The molds were positioned in succession and 100 mm apart, then fixed to the working surface with double-sided tape;

II. eight small plastic tubes were inserted along the fiber optic (between the FBGs and at the extremities) to strengthen the exposed portions of the fiber;

III. the fiber optic was inserted into the molds, passing through the lateral grooves and being careful to place each FBG at the midsection of the corresponding mold. The extremities of the fiber were then slightly pulled and secured with some adhesive tape to keep the FBGs properly tight.

IV. two strips of kinesio tape 2 mm wide (ALPIDEX, BB Sport GmbH & Co KG., Töging am Inn, Germany) were placed parallel to the fiber optic and fixed into the dedicate housings of each mold, in order to toughen the system;

V. part A and part B of a bi-component silicone rubber (Dragon SkinTM 30, Smooth-On, Inc., Macungie, PA, USA) were blended with a volume ratio of 1A:1B together with 10% in volume of liquid thinner (Silicone ThinnerTM, Smooth-On Inc., Macungie, PA, USA);

VI. the mixture was let degassing into a vacuum chamber for few minutes until the complete removal of air bubbles;

VII. the mixture was poured into each mold and let curing at room temperature for 24 hours;

VIII. once cured, the sensing elements were gently pulled out from the molds. Additional kinesio tape was used to cover the portions of the optical fiber which were not encapsulated into the polymeric substrates.

The steps of the fabrication process are represented in Figure S1.

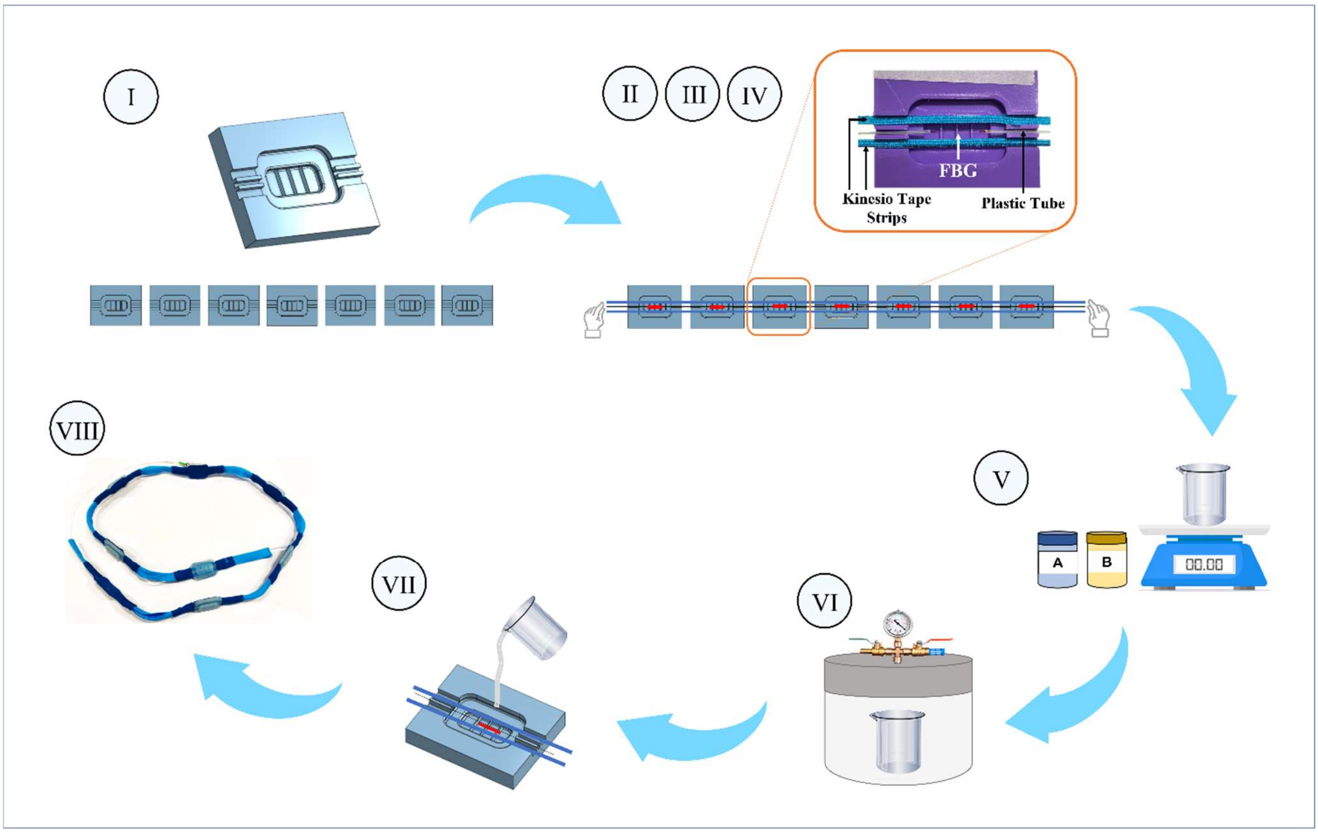

Figure S1. Representation of the eight steps composing the manufacturing process of wearable system.

2. The Sensing Elements' Response to Strain

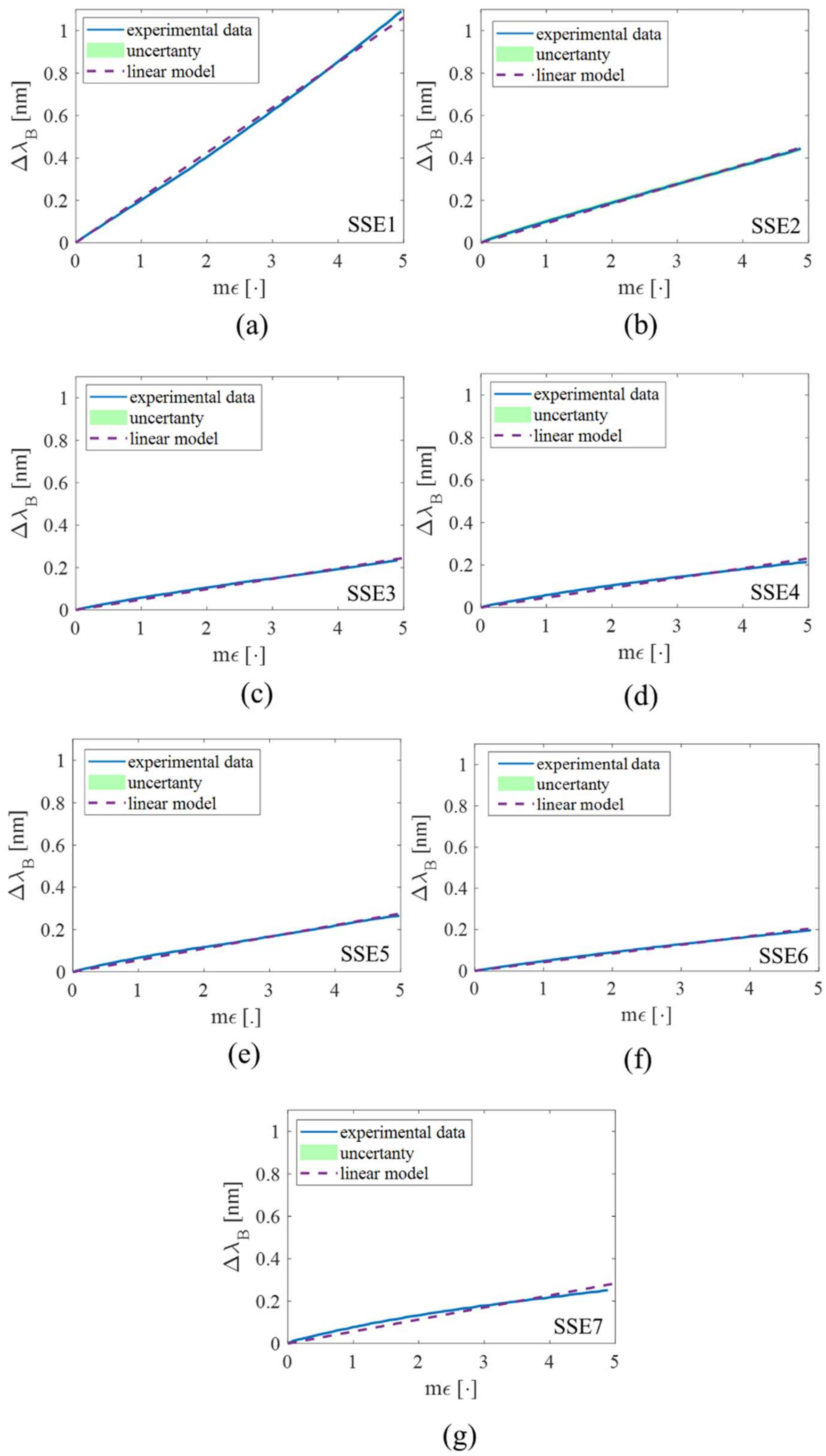

Figure S2. The calibration curve of $\Delta\lambda_B$ vs. mε of the seven SSEs: (**a**) SSE 1, (**b**) SSE 2, (**c**) SSE 3, (**d**) SSE 4, (**e**) SSE 5, (**f**) SSE 6 and (**g**) SSE 7. The mean experimental $\Delta\lambda_B$ signal is shown by the blue line, the uncertainty is shown by the shadowed green area, and the linear model is shown by the dotted purple line.

3. The Sensing Elements' Response to Temperature

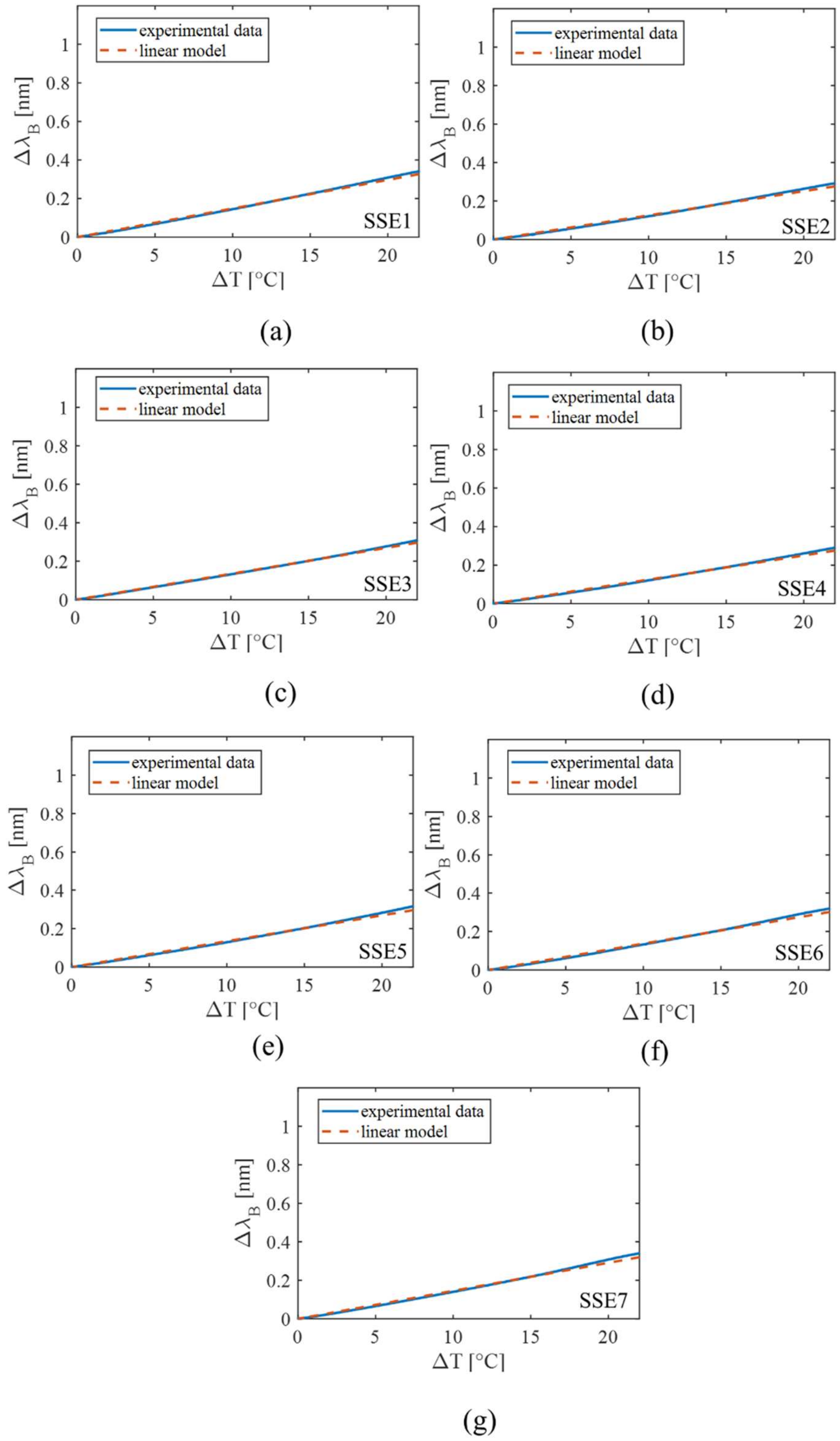

Figure S3. The calibration curve of $\Delta\lambda_B$ vs. ΔT of the seven SSEs: (**a**) SSE 1, (**b**) SSE 2, (**c**) SSE 3, (**d**) SSE 4, (**e**) SSE 5, (**f**) SSE 6 and (**g**) SSE 7. The experimental $\Delta\lambda_B$ signal is shown as the blue line, while the linear model is shown as the dotted purple line.
